# Utility of Fetal Cardiovascular Magnetic Resonance for Prenatal Diagnosis of Complex Congenital Heart Defects

**DOI:** 10.1001/jamanetworkopen.2021.3538

**Published:** 2021-03-29

**Authors:** Daniel Salehi, Katrin Fricke, Misha Bhat, Håkan Arheden, Petru Liuba, Erik Hedström

**Affiliations:** 1 Clinical Physiology, Department of Clinical Sciences Lund, Lund University, Skåne University Hospital, Lund, Sweden; 2 Pediatric Cardiology, Department of Clinical Sciences Lund, Lund University, Skåne University Hospital, Lund, Sweden; 3 Diagnostic Radiology, Department of Clinical Sciences Lund, Lund University, Skåne University Hospital, Lund, Sweden

## Abstract

**Importance:**

Prenatal diagnosis of complex congenital heart defects reduces mortality and morbidity in affected infants. However, fetal echocardiography can be limited by poor acoustic windows, and there is a need for improved diagnostic methods.

**Objective:**

To assess the clinical utility of fetal cardiovascular magnetic resonance imaging in cases in which fetal echocardiography could not visualize all relevant anatomy.

**Design, Setting, and Participants:**

This cohort study was conducted between January 20, 2017, and June 29, 2020, at Skåne University Hospital (Lund, Sweden), a tertiary center for pediatric cardiology and thoracic surgery. Participants were fetuses referred for fetal cardiovascular magnetic resonance examination by a pediatric cardiologist after an inconclusive echocardiograph.

**Exposures:**

Fetal cardiovascular magnetic resonance examination requested by the patient’s pediatric cardiologist.

**Main Outcomes and Measures:**

Any change in patient management because of diagnostic information gained from fetal cardiovascular magnetic resonance imaging.

**Results:**

A total of 31 fetuses underwent cardiovascular magnetic resonance examination at a median gestational age of 36 weeks (range, 31-39 weeks). Overall, fetal cardiovascular magnetic resonance imaging had clinical utility, affecting patient management and/or parental counseling in 26 cases (84%). For aortic arch anatomy including signs of coarctation (20 fetuses), fetal cardiovascular magnetic resonance imaging added diagnostic information in 16 cases (80%). For assessment of univentricular vs biventricular outcome in borderline left ventricle, unbalanced atrioventricular septal defect, and pulmonary atresia with intact ventricular septum (15 fetuses), fetal cardiovascular magnetic resonance imaging visualized intracardiac anatomy and ventricular function, allowing assessment of outcome in 13 cases (87%). In 4 fetuses with hypoplastic left heart syndrome, fetal cardiovascular magnetic resonance imaging helped delivery planning in 3 cases (75%). Finally, fetal cardiovascular magnetic resonance imaging provided valuable information for parental counseling in 21 cases (68%).

**Conclusions and Relevance:**

In this cohort study, fetal cardiovascular magnetic resonance imaging added clinically useful information to what was available from echocardiography. These findings suggest that fetal CMR has the potential to affect clinical decision-making in challenging cases of congenital heart defects with inconclusive data from echocardiography. Fetal cardiovascular magnetic resonance imaging showed an association with clinical decision-making, including mode of delivery and early postnatal care, as well as with parental counseling.

## Key Points

**Question:**

Does fetal cardiovascular magnetic resonance improve prenatal diagnosis of complex congenital heart defects when fetal echocardiography is insufficient?

**Findings:**

In this cohort study of 31 fetuses referred for fetal cardiovascular magnetic resonance imaging, additional clinically useful information regarding cardiovascular anatomy and function was obtained in 84% of referred cases, covering a wide spectrum of congenital heart defects. This information affected choice of mode of delivery, planning of early postnatal care, and parental counseling.

**Meaning:**

These findings suggest that fetal cardiovascular magnetic resonance imaging can add important diagnostic information and affect clinical decision-making and parental counseling.

## Teaser

This cohort study assesses the clinical utility of fetal cardiovascular magnetic resonance imaging when fetal echocardiography could not visualize all relevant anatomy.

## Introduction

Congenital heart defects (CHDs) affect approximately 1% of the population^1^ and account for approximately 30% of infant mortality related to congenital anomalies.^2^ Complex CHDs, with need of intervention within the first year of life, account for 25% of all CHD cases.^3^ Prenatal detection of complex CHD leads to decreased mortality and morbidity by improved delivery planning and immediate availability of adequate postnatal care, especially in duct-dependent lesions.^4,5,6,7,8^ Furthermore, improved prenatal diagnosis is important for parental counseling.^9^ Although prenatal diagnosis rates have improved during the last decades, the overall detection rate of major CHD is 70%, with a wide range as low as 20% for coarctation of the aorta.^10^ Furthermore, detection rates vary highly between countries and centers. Thus, there is a need for improved imaging methods for prenatal diagnosis of cardiovascular malformations.

Until recently, fetal cardiovascular magnetic resonance (CMR) imaging has been dependent on either real-time acquisition, which typically is of low spatial resolution, static acquisition, or in-house postprocessing techniques due to lack of methods for direct cardiac gating.^11,12,13,14,15,16^ With the development of an MR-compatible Doppler ultrasound (DUS) device,^17^ it is possible to acquire high-resolution fetal CMR images without postprocessing, making fetal CMR potentially more clinically applicable.^18,19,20,21^ Since echocardiography requires less time and resources compared with CMR, fetal CMR would likely serve as a complement to fetal echocardiography in challenging cases. Previous studies on the clinical utility of fetal CMR have shown that it can be used as an adjunct to echocardiography in cases with insufficient information from ultrasound.^22^ However, these studies focused on extracardiac vascular anomalies^23^ or relied on real-time acquisition with limited spatial resolution for cardiac cine imaging.^16^ Standard and widely available fetal cardiac-gated cine sequences may increase visualization of intracardiac anatomy and cardiac function and increase image quality of the fetal heart and large vessels. However, it is unknown to what extent this may affect clinical care. Therefore, the aim of this study was to evaluate the added diagnostic value and potential effect on clinical care using fetal CMR imaging in cases of suspected CHD with incompletely visualized cardiovascular anatomy and function by fetal echocardiography.

## Methods

Patients were included at Skåne University Hospital, Lund, Sweden, which is 1 of 2 tertiary centers in Sweden for pediatric cardiothoracic surgery, with approximately 300 fetal echocardiography examinations performed each year. Patients in the current study were included between January 20, 2017, and June 29, 2020. All participants provided written informed consent. The regional ethics committee in Lund, Sweden, approved the study. The study was conducted in accordance with the Helsinki declaration.^24^ The Strengthening the Reporting of Observational Studies in Epidemiology (STROBE) reporting guideline was used as a guideline for reporting the current study.

All study participants underwent routine ultrasound screening, during which a suspicion for CHD was raised. Patients were subsequently referred to our tertiary center for at least 1 fetal echocardiography, performed by a pediatric cardiologist specialized in fetal echocardiography and with extensive experience. If fetal echocardiography was insufficient for diagnosis, the pediatric cardiologist referred the patient for fetal CMR and inclusion in the current study. The CMR protocol at 1.5 T (Siemens Aera) consisted of anatomical balanced steady state free precession (bSSFP) images in the sagittal, transversal, and coronal planes; cardiac cine images in a transversal stack or left ventricular short-axis stack and standard long-axis views (4-chamber, 3-chamber, and 2-chamber views); and cine images of the aortic arch, pulmonary artery, and arterial duct. Cine image acquisitions were acquired using either DUS gating (northh medical)^18^ or, when a DUS signal was not available, self-gating by tiny golden angle iterative golden-angle radial sparse parallel (iGRASP).^14,15^ Anatomical bSSFP images were acquired as a stack covering the heart and great vessels with a typical acquired resolution of 1.7 × 1.1 × 4.5 mm^3^, with a slice gap of 0% or –50% (ie, overlapping slices). Cine imaging by DUS gating was set to a typical acquired spatial resolution of 1.4 × 1.4 × 4 mm^3^ and an acquired temporal resolution of 38 milliseconds per frame; iGRASP was set to a spatial resolution of 1.6 × 1.6 × 4 mm^3^ and temporal resolution of 29 milliseconds. If needed, cine images of the aortic arch, pulmonary artery, and arterial duct were acquired as an image stack with overlapping slices (slice gap, –50%). In fetuses with hypoplastic left heart syndrome with suspected restrictive or intact atrial septum and in fetuses with suspected obstructed total anomalous venous return, a transversal T2-weighted stack (echo time, 143 milliseconds; repetition time, 1250 milliseconds; acquired spatial resolution, 1.3 × 1.1 × 5 mm^3^; receiver bandwidth, 64 kHz) covering the thorax was acquired to determine whether pleural effusion and the so-called nutmeg lung pattern were present, which would be indicative of pulmonary lymphangiectasia associated with poor postnatal outcome.^25^ Images were preferably acquired in free breathing to simplify the procedure for the pregnant woman, whereas breath-hold images were used if free-breathing images were of insufficient quality.

Fetal CMR images were evaluated by 3 observers in consensus (D.S., K.F., and E.H., or D.S., M.B., and E.H.), all together with extensive experience in fetal cardiology, echocardiography, and CMR and who also had access to echocardiography images and reports. Fetal CMR was considered associated with patient management if additional diagnostic information from fetal CMR affected clinical decision-making. The association of information from fetal CMR with prenatal counseling was evaluated by the pediatric cardiologist (K.F. or M.B.). Improvement was considered if fetal CMR was able to provide a more definitive diagnosis to parents and more accurate expectations regarding postnatal management and course. For the purposes of this study, fetal CMR findings and assessments were compared with postnatal echocardiography findings and postnatal outcomes.

### Statistical Analysis

No statistical testing was conducted for this study. Data were collected and organized using Excel 365 (Microsoft Corp).

## Results

**Figure 1.**
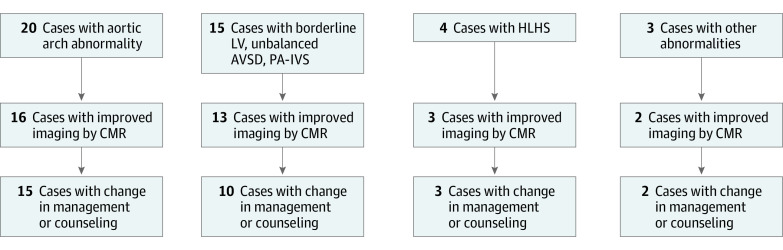
Overview of the Study Population and Utility of Fetal Cardiovascular Magnetic Resonance (CMR) A flowchart of the included main groups of suspected pathology, in how many cases fetal CMR led to improved imaging and information, and change in management or counseling. AVSD indicates atrioventricular septal defect; LV, left ventricle; HLHS, hypoplastic left heart syndrome; PA-IVS, pulmonary atresia with intact ventricular septum.

Overall, 31 fetuses underwent fetal CMR at gestational age 36 (31–39) weeks. Scan duration was approximately 20 to 40 minutes, the latter including reacquisition of images because of fetal motion. Figure 1 shows a flowchart of the included groups of suspected pathology, the number of cases for which fetal CMR led to improved imaging, and change in management or counseling. The eTable in the Supplement provides more details regarding the suggested prenatal diagnosis by fetal echocardiography, CMR requests by the referring physician, information obtained by CMR, and how this compared with postnatal diagnosis and outcome. Fetal CMR affected patient management and/or parental counseling in 26 cases (84%). In the remaining 5 cases, no diagnostic questions were clarified by fetal CMR.

The 4 main, nonexclusive categories for referral to fetal CMR were (1) to determine aortic arch anatomy including isthmus anatomy; (2) to measure atrioventricular valve annulus diameter and to evaluate ventricular size, function, and morphology for assessment of univentricular vs biventricular outcome in cases of borderline left ventricle, pulmonary atresia with intact ventricular septum, or unbalanced atrioventricular septal defect; (3) to assess signs of restrictive atrial septum, including pulmonary lymphangiectasia in cases of hypoplastic left heart syndrome, and suspected restrictive or intact atrial septum for risk stratification and planning of delivery, including deciding on cesarean or vaginal delivery, and whether to have the cardiac catheterization laboratory on standby at the time of delivery; and (4) to provide information for parental counseling regarding diagnosis, postnatal outcome, possible mode of surgery, and postnatal care (Figure 1; eTable in the Supplement).

**Figure 2.**
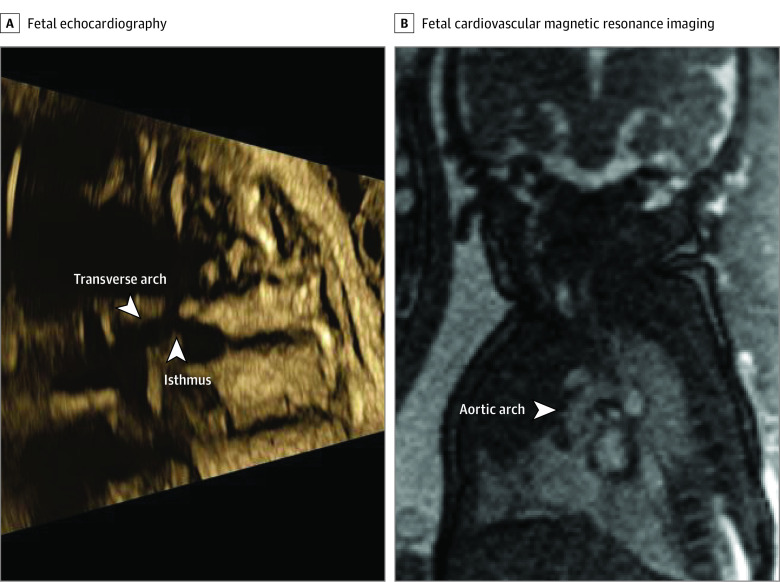
Evaluation of Aortic Arch Anatomy in a Fetus With Suspected Aortic Arch Hypoplasia and/or Coarctation Fetal echocardiography (A) showed suspected aortic arch hypoplasia and/or coarctation. The arrows indicate a narrow transverse aortic arch and aortic isthmus. However, fetal cardiovascular magnetic resonance imaging showed a normal-sized aortic arch with no signs of coarctation (B). The CMR findings were confirmed by postnatal echocardiography (not shown). Contrast and brightness in the CMR image have been optimized for printing (case 8; eTable in the Supplement).

The first category (aortic arch anatomy) included 20 cases (cases 3, 6-10, 12-13, 18-27, 28, 31) (eTable in the Supplement). Fetal CMR provided additional diagnostic information in 16 of those 20 cases (80%) (Figure 2). In the remaining 4 cases, fetal CMR image quality was insufficient. In 4 of the 16 cases (25%) in which additional diagnostic information was obtained, fetal CMR showed normal aortic and cardiac anatomy, dismissing the suspicion of aortic coarctation.

**Figure 3.**
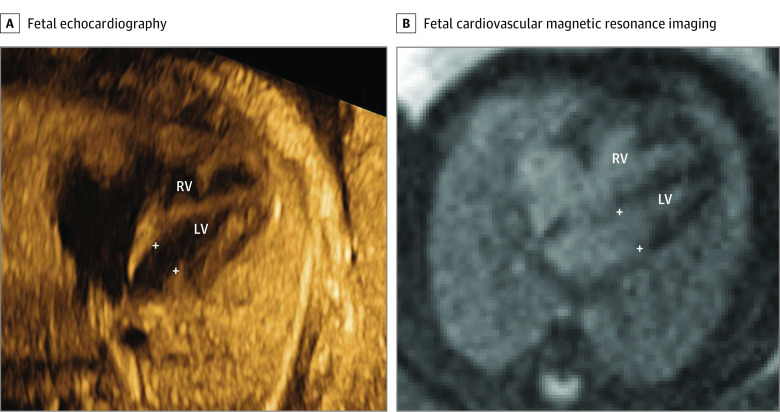
Measurement of Atrioventricular Valve Annulus for Assessment of Univentricular vs Biventricular Outcome A 4-chamber image by fetal echocardiography (A) showed an underdeveloped mitral valve annulus (5.6 mm; *z* score, −3.2) and a non–apex-forming left ventricle; however, the case was suspected to be underestimated or misdiagnosed due to suboptimal image projection. A 4-chamber image by fetal cardiovascular magnetic resonance (B) showed a normal-sized mitral valve annulus (9.4 mm; *z* score −0.3), 2 papillary muscles, and a narrow, almost apex-forming, left ventricle (LV) with normal function, suggesting biventricular outcome, as confirmed postpartum. White crosses indicate the mitral annulus. Contrast and brightness in the CMR image have been optimized for printing (case 13) (eTable in the Supplement). RV indicates right ventricle.

The second category (assessment of biventricular vs univentricular outcome) included 15 cases (cases 3-4, 6, 8, 13-17, 20, 24-27, 29) (eTable in the Supplement). In all cases, estimation of atrioventricular valve annulus size was requested by the referring physician. Cine images of the left and right ventricles allowed for assessment of ventricular function and morphology in all cases. An example of cine image quality is shown in the Video. Fetal CMR could visualize the atrioventricular valve annulus, allowing for assessment of outcome in 13 of these cases (87%) (Figure 3). In 1 case in which fetal CMR provided additional information, the mitral valve was severely underdeveloped and left ventricular lateral wall contractility reduced. In this case, there was uncertainty regarding outcome despite adequate visualization of ventricular anatomy and function. Among the remaining 12 cases, the assessment of biventricular or univentricular outcome was correct in 11 cases (92%).

**Video.** Cardiovascular Magnetic Resonance (CMR) Imaging for Prenatal Diagnosis of Complex Congenital Heart Defects CMR cine image of a fetal heart in the 4-chamber view. Fetal ultrasonography suggested underdeveloped mitral valve annulus and a left ventricle with suspected aortic arch hypoplasia and hypoplastic aortic isthmus. CMR imaging showed a mildly underdeveloped LV with normal function. On delivery, the infant was found to have aortic coarctation. For details, see case 13 in the eTable in the Supplement.

**Figure 4.**
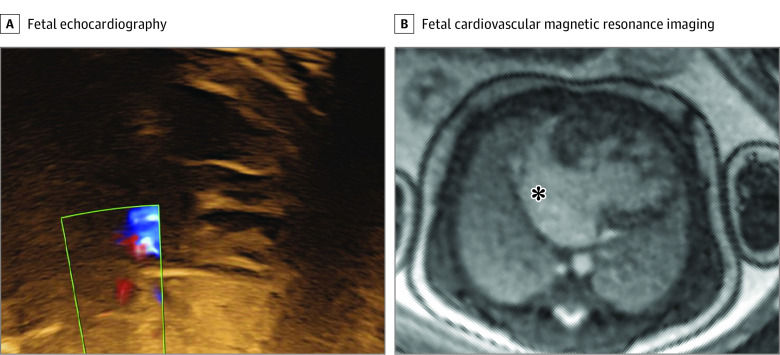
Evaluation of Atrial Restriction in a Fetus With Hypoplastic Left Heart Syndrome Fetal echocardiography could not visualize the atrial cavity or Doppler flow across the atrial septum due to poor acoustic windows (A). Fetal cardiovascular magnetic resonance showed a large interatrial communication, indicated by the asterisk, and no nutmeg pattern (B). Therefore, the risk of restrictive atrial septum was considered low (albeit a membrane could not be ruled out), and the fetus was planned for a vaginal delivery without cardiac catheterization laboratory on standby. Contrast and brightness in the CMR image have been optimized for printing (case 5) (eTable in the Supplement).

The third category (hypoplastic left heart syndrome with suspected restrictive or intact atrial septum) included 4 fetuses (cases 5, 11, 28, 31) (eTable in the Supplement). In 1 case the reason for fetal CMR referral was hypoplastic left heart syndrome with unclear interatrial communication due to poor acoustic windows in late gestation. Fetal CMR visualized the fetal heart with a large interatrial communication and no signs of pulmonary lymphangiectasia. This changed the delivery planning from cesarean delivery with catheterization laboratory on standby to vaginal delivery without catheterization laboratory on standby (Figure 4). In a similar case, fetal CMR showed an adequate interatrial communication and no signs of pulmonary lymphangiectasia, leading to a similar change in clinical management. The third case of hypoplastic left heart syndrome included in the current study showed echocardiographic signs of restrictive atrial septum. Fetal CMR showed no signs of pulmonary lymphangiectasia. Delivery was planned for cesarean delivery with potential access to intervention. A restrictive atrial septum was confirmed postnatally, without signs of pulmonary vascular disease, and the patient was stable enough to undergo a Norwood procedure on day 1. In the fourth case, fetal echocardiography showed a univentricular heart, and hypoplastic left heart syndrome was suspected. Acoustic windows were poor due to the mother having obesity, and neither the atrial septum nor the connection of pulmonary veins or great arteries to the heart could be visualized. Fetal CMR confirmed the diagnosis of hypoplastic left heart syndrome and showed an adequate interatrial communication as well as normal connection of at least 2 pulmonary veins to the left atrium and ventriculo-arterial concordance. The information from fetal CMR in this case changed delivery planning from cesarean delivery with catheterization laboratory on standby to vaginal delivery without catheterization laboratory on standby.

The fourth category (complex CHD and parental counseling) included 21 fetuses (cases 2-4, 6, 8, 11-13, 16, 18, 19-21, 23-29, 31) (eTable in the Supplement). In these cases, parental counseling was improved regarding information about the congenital heart defect of the fetus, discussion of treatment options, and postnatal outcome.

**Figure 5.**
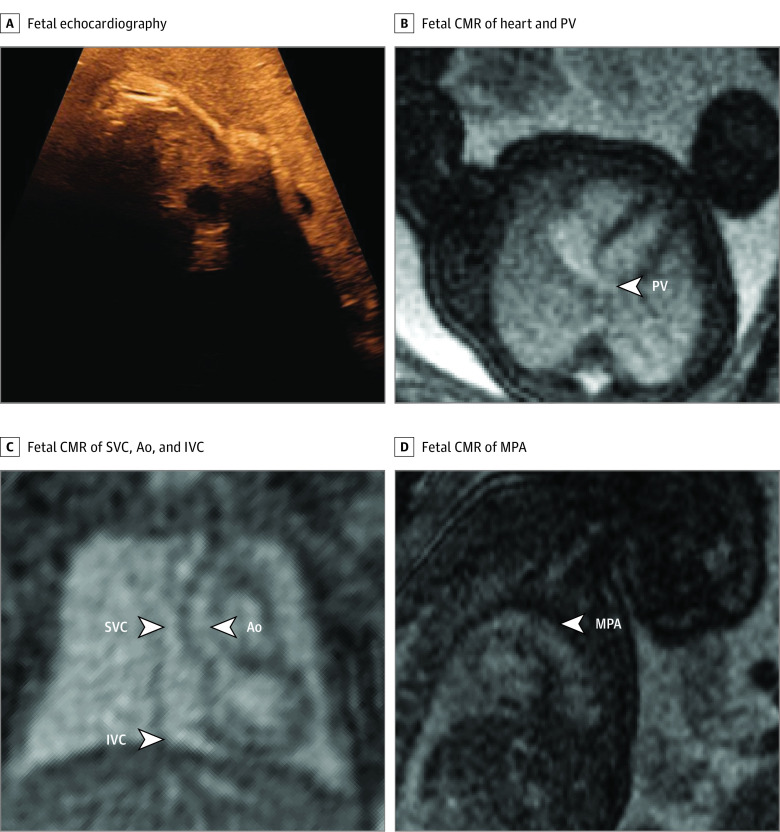
Basic Assessment in a Fetus With Risk Factors for Cardiac Malformation and a Very Poor Acoustic Window The fetal heart and vessels were not visible at all during fetal echocardiography, due to mother having obesity (A). Fetal cardiovascular magnetic resonance imaging (MRI) showed normal-sized ventricles with normal systolic function and at least 1 normal pulmonary vein (PV) without obvious direct or indirect signs of aberrant pulmonary veins (B), superior venae cavae (SVC) and inferior venae cavae (IVC) and normal ascending aorta (Ao) (C), and normal main pulmonary artery (MPA) (D). This information ruled out major congenital heart disease, and delivery was planned at the hospital closest to the patient’s home. A nonurgent postnatal echocardiography examination was performed before discharge, which showed a normal cardiovascular anatomy. Contrast and brightness in the CMR images have been optimized for printing (case 30) (eTable in the Supplement).

In 1 case of particular interest with regard to the current limitations of fetal echocardiography, the mother having obesity hampered diagnosis by echocardiography in a pregnancy with multiple risk factors for CHD because the fetal heart was not visible at all (Figure 5). In this case, fetal CMR demonstrated normal-sized left and right ventricles with normal systolic function, no inflow anomalies, no anomalies of outflow tracts, no anomalies of great vessels, at least 1 visible pulmonary vein to the left atrium, and no obvious direct or indirect signs of aberrant pulmonary veins. These findings allowed for delivery at the hospital closest to the patient’s home with nonurgent postnatal evaluation. Postnatal echocardiography confirmed normal cardiovascular anatomy.

Fetal CMR provided information that did not agree with postnatal diagnosis and/or clinical course in 2 cases. These cases were related to assessment of postnatal univentricular vs biventricular outcome and postnatal development of aortic coarctation, respectively (cases 13 and 14) (eTable in the Supplement).

## Discussion

This study suggests that fetal CMR is a useful complement to fetal echocardiography for evaluating cardiac function and both intracardiac and vascular anatomy in a wide spectrum of CHDs. In 84% of cases referred for CMR due to uncertainties after echocardiography, CMR added clinically useful information that affected planning of delivery, postnatal care, and/or parental counseling.

This study adds the perspective of clinical utility of fetal cardiac-gated cine CMR for cardiac function and intracardiac assessment, whereas earlier studies focused on methods development^14,18,26^ or mainly vascular assessment or real-time acquisition with limited spatial resolution for cine imaging.^16,20,22,23^ In the current study, fetal CMR affected delivery planning (ie, vaginal vs cesarean delivery, delivery at tertiary hospital vs at the hospital closest to the patient’s home) and postnatal care (ie, urgent vs nonurgent postnatal clinical evaluation, need vs no need of immediate prostaglandin infusion, whether to have the cardiac catheterization team prepared for immediate intervention). Furthermore, increased knowledge from fetal CMR in challenging cases provided valuable information, allowing for improved parental counseling.

In patients referred for fetal CMR for evaluation of aortic arch morphology, added diagnostic information was obtained in 16 of 20 cases (80%). However, in some cases, the entire aortic arch was challenging to visualize by CMR. Cine CMR images of the aortic arch provided high-quality images of aortic anatomy, confirming the role of cardiac-gated sequences for fetal vascular imaging.^20^ In cases in which cine imaging of the aorta was challenging due to acquisition time, fetal movements, or an unstable DUS signal for gating, fast nongated bSSFP or iGRASP sequences could occasionally provide diagnostic information. In fetuses with high suspicion of postnatal development of coarctation of the aorta due to aortic arch anomalies and/or moderately underdeveloped left-sided cardiac structures, delivery in a tertiary center including admission to the intensive care unit, prompt examination by a pediatric cardiologist, and administration of prostaglandins were recommended. In fetuses with low prenatal suspicion of coarctation of the aorta, delivery usually took place at the tertiary center, and the newborn was examined within 72 to 96 hours before discharge. In cases in which fetal CMR showed a normal aortic arch morphology with a normal-sized aortic arch and isthmic region, fetal coarctation of the aorta could be ruled out, and the child was delivered at the hospital closest to the patient’s home.

In the current study, fetal CMR provided measurements of atrioventricular valve annulus size and assessed ventricular function and morphology in patients with borderline left ventricle, pulmonary atresia with intact ventricular septum, and atrioventricular septal defect when ultrasound was insufficient for assessment of outcome. Fetal CMR provided additional diagnostic information and made a difference for prenatal assessment of univentricular vs biventricular outcome. In cases of borderline left ventricle, there is a risk of acute left-sided heart failure soon after birth related to the underdeveloped left-sided cardiovascular structures. Therefore, prenatal risk assessment is crucial in these cases for planning of delivery and immediate postnatal care, but it remains challenging. Several models to predict risk of left ventricular failure after birth have been developed. Although they consider both anatomical and functional features of the fetal heart, they are far from perfect.^27,28^ To what extent fetal CMR may further improve prenatal assessment of univentricular vs biventricular outcome in borderline left ventricle remains to be shown.

Fetal CMR was incorrect in assessing postnatal outcomes in 2 cases. In all fetuses with unbalanced atrioventricular septal defect in the current study, fetal CMR provided measurement of atrioventricular annulus size indicative of biventricular outcome. However, in 1 of those fetuses biventricular outcome was not possible due to a structural anomaly of the left atrioventricular valve that was unknown before birth. Likely, CMR could not visualize this due to limited resolution of current fetal CMR sequences, indicating a need for further improvement of spatial resolution. In another case, fetal CMR was incorrect regarding whether aortic coarctation would develop. Although commonly applied, neither atrioventricular annulus measurements nor ventricular morphology are certain factors for whether biventricular circulation will succeed postnatally nor is size or morphology of the aortic isthmus a certain biomarker for the development of postnatal coarctation.^27,28,29,30,31,32,33^ These uncertainties may in themselves explain different postnatal outcomes compared with prenatal assessment, as previously known from ultrasound. Although fetal cardiac anatomy presumably was adequately visualized by CMR, as noted in image quality in the current study, new biomarkers for outcomes are needed to improve prenatal assessments. Future fetal CMR could potentially provide such biomarkers by quantitative measures of physiology beyond what is possible today.

In fetuses with hypoplastic left heart syndrome where structures of interest could not be visualized by echocardiography, fetal CMR affected the mode of delivery and planning of early postnatal intervention. Fetal CMR allowed visualization of the atrial septum and interatrial communication and assessed presence of the so-called nutmeg lung pattern as a sign of pulmonary lymphangiectasia. Although the sensitivity of prenatal nutmeg lung pattern for diagnosing pulmonary lymphangiectasia is uncertain, this finding is associated with high postnatal mortality in patients with hypoplastic left heart syndrome with restrictive atrial septum.^25^ Survival after birth is dependent on the right ventricle and a left-to-right atrial shunt and a patent arterial duct.^34^ In cases with restrictive or intact atrial septum when an atrial shunt is insufficient or not present, the newborn needs immediate cardiac intervention.^34,35,36^ In such cases, planned delivery by cesarean section with catheterization laboratory personnel on standby for immediate intervention is crucial. However, if pulmonary lymphangiectasia is present in such a case, survival of the infant is unlikely, and vaginal delivery may instead be chosen to not subject the pregnant woman to unnecessary risk of cesarean delivery.

Beyond providing a more definitive diagnosis to parents and more accurate expectations regarding postnatal management and course, fetal CMR allowed parents to feel comfortable with the choice of delivery center, whether it was an advanced tertiary care hospital or their local hospital. In high-risk hypoplastic left heart syndrome with restrictive atrial septum, ruling out pulmonary lymphangiectasia provided a more favorable prognosis, offering hope to parents, and was an important piece of information in the decision of recommending caesarian delivery with the operating room on standby.

### Limitations

This study has limitations. It included third-trimester fetuses because current CMR methods are best suited for relatively large fetuses, in which gross fetal movement is small and fetal cardiovascular structures are large enough to be visualized with the currently available spatial resolution. Understanding the complexity of CHD earlier in pregnancy is crucial for counseling and pregnancy decision-making, and there is a need for improved fetal cardiovascular imaging earlier in pregnancy. Recent motion-corrected fetal CMR methods may improve image quality during earlier stages of pregnancy but are not yet widely available. There are no CMR-based reference values for fetal cardiovascular anatomy, so echocardiography-based reference values were applied. Although likely small, there may be a bias between measurements by fetal CMR and echocardiography that could confound measurements vs outcome assessment using echocardiography-based reference values. Also, different echocardiography-based reference values differ highly. Future studies are needed to provide CMR-based reference values for increased accuracy. Furthermore, the comparison of prenatal CMR with postnatal echocardiography functional parameters and size measurements of cardiovascular structures may be confounded by the postnatal transition of circulation, including redistribution of volumes and blood flow.

## Conclusions

In this study, fetal CMR added clinically useful information to echocardiography in referred cases and was a useful complement to fetal echocardiography for evaluating cardiac function and both intracardiac and vascular anatomy in CHDs. Fetal CMR affected clinical decision-making, including mode of delivery and early postnatal care, as well as parental counseling.

## Notes

Supplement. **eTable. **Clinical Cases of Fetuses With Suspected Complex Congenital Heart Defects Referred for Fetal CMR
