# Errors in Figure Titles and Article Information

**DOI:** 10.1001/jamanetworkopen.2021.11261

**Published:** 2021-04-23

In the Original Investigation titled “Utility of Fetal Cardiovascular Magnetic Resonance for Prenatal Diagnosis of Complex Congenital Heart Defects,”^1^ published March 29, 2021, there were errors in the titles of Figures 2 and 5 and in the Author Contributions section. The title of Figure 2 should have read “Evaluation of Aortic Arch Anatomy in a Fetus With Suspected Aortic Arch Hypoplasia and Coarctation,” and the titles for Figures 5B, 5C, and 5D should have included “Fetal CMR.” In the Author Contributions section, the authors listed under Supervision should have been Arheden, Liuba, and Hedström. This article has been corrected.^1^
