# Author Name Change

**DOI:** 10.1001/jamanetworkopen.2022.5825

**Published:** 2022-03-15

In the Original Investigation titled “Utility of Fetal Cardiovascular Magnetic Resonance for Prenatal Diagnosis of Complex Congenital Heart Defects,”^1^ published March 29, 2021, the first author’s last name changed after publication from Salehi to Ryd. The article was previously corrected to fix errors in the titles of Figures 2 and 5 and in the Author Contributions section.^2^
