# Supplementary material for: Utility of Fetal Cardiovascular Magnetic Resonance for Prenatal Diagnosis of Complex Congenital Heart Defects
Source: JAMA Netw Open. 2021 Mar 29;4(3):e213538. doi: 10.1001/jamanetworkopen.2021.3538 (PMC8008290; doi:10.1001/jamanetworkopen.2021.3538)
Supplement: Supplement. — eTable. Clinical Cases of Fetuses With Suspected Complex Congenital Heart Defects Referred for Fetal CMR [file jamanetwopen-e213538-s001.pdf]

Supplemental Online Content

Salehi D, Fricke K, Bhat M, Arheden H, Liuba P, Hedström E. Utility of fetal cardiovascular magnetic resonance for prenatal diagnosis of complex congenital heart defects. *JAMA Netw Open*. 2021;4(3):e213538. doi:10.1001/jamanetworkopen.2021.3538

eTable. Clinical Cases of Fetuses With Suspected Complex Congenital Heart Defects Referred for Fetal CMR

This supplemental material has been provided by the authors to give readers additional information about their work.

© 2021 Salehi D et al. *JAMA Network Open.*

eTable 1. Clinical Cases of Fetuses With Suspected Complex Congenital Heart Defects Referred for Fetal CMR

Patient	Prenatal echocardiography diagnosis	Remaining diagnostic questions to be clarified by fetal CMR	What fetal CMR answered to remaining diagnostic questions	Postnatal diagnoses	CMR was correct compared with postnatal diagnoses	Impact of fetal CMR on patient care
1	- Situs inversus.<br>- Pulmonary atresia.<br>- Unbalanced AVSD.<br>-Interrupted IVC.<br>- Bowel obstruction.	1. Infracardiac TAPVR?<br>2. Pulmonary lymphganciectasia?<br>3. Confirmation of systemic venous anomalies.	1. Not visualized (extensive fetal movement; polyhydramniosis).<br>2. No pulmonary lymphangiectasia or signs of severe obstruction.<br>3. Not visualized.<br>- Duodenal obstruction.	- Mildly obstructed TAPVR.<br>- Pulmonary atresia.<br>- Unbalanced AVSD.<br>- Dextrocardia with situs inversus.	1. –<br>2. Yes<br>3. –	Due to suspicion of already poor prognosis, an additional diagnosis of pulmonary lymphangiectasia would have led to possible delivery at hospital near home and a plan for primary palliative care would

© 2021 Salehi D et al. *JAMA Network Open.*

	- Suspected ipsilateral hepatic veins and TAPVR.			- Extracardiac malformations (bowel obstruction).		have been considered. Since no lymphangiectasia was identified, patient was delivered at tertiary care center.
2	- TGA/VSD-PS (suboptimal acoustic windows)	1. Measurement of pulmonary valve annulus to determine if arterial switch operation vs Rastelli to be performed.	1. Mildly underdeveloped pulmonary valve annulus.	- Valvular PS.<br>- TGA<br>- VSD	1. Yes	- Rastelli performed.<br>- No change in patient management but improved counseling.
3	- Borderline LV (suboptimal projection of MV annulus).<br>- VSD	1. Aortic arch anatomy (hypoplasia, isthmus size).<br>2. MV annulus size (uni- vs biventricular outcome?).	1. Aortic arch not visualized.<br>2. MV annulus slightly underdeveloped (z score -2.7).	- LV slightly hypoplastic with normal function.<br>- MV annulus slightly	1. –<br>2. Yes	Counseling concerning likelihood of biventricular outcome, no further information

© 2021 Salehi D et al. *JAMA Network Open.*

	- Aortic arch hypoplasia? (no sagittal view by echocardiography)		→ Biventricular repair may be possible. Unable to assess risk of CoA.	underdeveloped (z score -2.3).<br>- Aortic arch hypoplasia and CoA.<br>(Biventricular outcome)		concerning arch anatomy.<br><br>No change in patient management.
4	- Aortic stenosis.<br>- Aortic arch hypoplasia.<br>- Borderline LV with small MV annulus and LV size.<br>(suboptimal projection)	1. MV annulus size?<br>2. LV size and function?<br>(Uni- vs biventricular outcome?)	1. MV annulus normal size (z score -1.7).<br>2. Narrow, almost apex-forming LV with good function.<br><br>→ Likely biventricular repair.	- Aortic stenosis.<br>- CoA<br>- No aortic arch hypoplasia.<br>- Well functioning LV.<br>- Normal-sized MV annulus.	1. Yes<br>2. Yes	Counseling regarding high probability of biventricular outcome.

© 2021 Salehi D et al. *JAMA Network Open.*

				(Biventricular outcome)		
5	- HLHS (difficult to visualize intracardiac anatomy)	1. Restrictive atrial septum?<br>2. Lymphangiectasia?	1. Large interatrial communication.<br>2. No lymphangiectasia.	- HLHS with large interatrial communication.	1. Yes<br>2. Yes	Standard-risk HLHS: vaginal delivery without cath lab on standby.
6	- Borderline LV with small MV annulus (suboptimal projection).<br>- Unclear if arch hypoplasia/CoA	1. MV annulus size (uni- vs biventricular outcome?).<br>2. Aortic arch anatomy? (hypoplasia/size isthmus aortae).	1. MV annulus slightly underdeveloped (z score -2.4).<br>2. Arch hypoplasia (transverse arch z score -3.4). Isthmic hypoplasia (z score sagittal view -2.7, 3VT -	- CoA<br>- Moderate aortic arch hypoplasia.<br>- Perimembranous VSD of moderate size.<br>- Slightly underdeveloped left-sided cardiac	1: Yes<br>2: Yes	Counseling regarding high probability of biventricular outcome and need of CoA repair.<br><br>Vaginal delivery in tertiary center with

© 2021 Salehi D et al. *JAMA Network Open.*

	(no sagittal view of aortic arch obtained).		3.5). Aortic valve annulus normal-sized.<br><br>→ Likely aortic arch surgery. Likely biventricular repair.	structures. (Biventricular outcome)		prostaglandins (standby).
7	- Suspected vascular ring (right aortic arch and left arterial duct).<br>- However aberrant right subclavian artery not visualized.	1. Double aortic arch?	1. Right-sided arch visualized as well as left-sided duct. No visualized left arch.	- Double aortic arch with dominant right arch, diminutive left arch with atretic portion of posterior left arch.<br>- Left-sided duct.	1. No (However, this variant of double aortic arch with atresia of the left posterior arch and a left-sided arterial duct is	No

© 2021 Salehi D et al. *JAMA Network Open.*

	- No sagittal aortic arch view obtained.				challenging to differentiate from a right aortic arch with a left-sided arterial duct).	
8	Suspicion of aortic valve stenosis with stiff, echogenic left ventricular myocardium.<br><br>Slightly underdeveloped	1. Aortic arch anatomy?<br>2. Aortic stenosis (annulus, morphology, jet)?<br>3. LV morphology and function? (uni- vs biventricular outcome?)	1. No arch hypoplasia or CoA.<br>2. Near normal aortic annulus size, central jet visualized – cannot rule out mild stenosis.<br>3. Prominent LV wall. LV nearly apex-forming with good function.	- Mildly underdeveloped MV annulus.<br>- Mildly underdeveloped bicuspid aortic valve without stenosis.	1. Yes<br>2. Yes<br>3. Yes	Improved counseling regarding good chance for no need of intervention.

© 2021 Salehi D et al. *JAMA Network Open.*

	isthmic region (suspected CoA).		→ May not require postnatal intervention.	- No arch hypoplasia or CoA.<br>- Echogenic LV endocardium.<br>- LV nearly apex-forming.<br>- No need for surgery.<br>(Biventricular outcome)		
9	- DILV<br>- TGA<br>- Poor visualization of	1. Subaortic stenosis (duct dependent)?<br>2. Aortic arch size?	1. Subaortic area not visualized.<br>2. No suspicion of CoA.	- DILV<br>- TGA<br>- No subaortic stenosis.<br>- No CoA.	1. –<br>2. Yes	Prostaglandins not administered prior to first echo.

© 2021 Salehi D et al. *JAMA Network Open.*

	subaortic region and arch.					
10	- VSD<br>- Slight ventricular disproportion.<br>- No aortic arch hypoplasia but suspected narrowing in isthmic region with flow acceleration (CoA?).	1. CoA?	1. Ascending aorta normal-sized. Difficult to visualize the transverse aortic arch and isthmus.<br><br>→ Unable to assess CoA.	- No CoA or arch hypoplasia.	1. –	No change in patient management.
11	- HLHS with echocardiographic suspicion of RAS	1. Pulmonary lymphangiectasia?	1. No pulmonary lymphangiectasia.	- HLHS<br>- RAS without significant clinical	1. Yes	Cesarean delivery recommended with cath lab on standby. If

© 2021 Salehi D et al. *JAMA Network Open.*

	(pathologic PV Doppler and pathologic hyperoxygenation test with decreased reactivity).			pulmonary disease.		lymphangiectasia had been seen, vaginal delivery with no standby would be recommended. Important for counseling regarding prognosis.
12	- Slight ventricular disproportion.<br>- No arch hypoplasia.<br>- Suspected slight narrowing in isthmus.	1. CoA?	1. No CoA.	- No aortic arch hypoplasia.<br>- No CoA.	1. Yes	Counseling + risk stratification.<br><br>Delivery at hospital closest to patient's home.

© 2021 Salehi D et al. *JAMA Network Open.*

13	- Borderline LV with underdeveloped MV annulus (suboptimal projection).<br>- Suspected aortic arch hypoplasia and hypoplastic aortic isthmus.	1. MV annulus diameter?<br>2. LV size and function? (Uni- vs biventricular outcome?)<br><br>3. Aortic arch anatomy?	1. Normal-sized MV annulus (z score -0.8).<br>2. Good LV function.<br>→ Likely biventricular outcome. Likely no need of aortic arch intervention.<br><br>3. No aortic arch hypoplasia or CoA.	-Mildly underdeveloped left-sided cardiac structures.<br>- No aortic arch hypoplasia.<br>- CoA<br><br>(Biventricular outcome)	1. Yes<br>2. Yes<br>3. No	Counseling regarding biventricular outcome and low risk for postnatal CoA. However, CoA did develop (false safety?).
14	- Isomerism<br>- Dextrocardia<br>- Unbalanced AVSD favouring the right.	1. Diameter of the left component of the common AV valve?<br>2. LV size<br>(uni- vs biventricular outcome?)	1. Left component of the common AV valve z score -2.3).<br>2. LV almost completely apex-forming.	- Unbalanced AVSD.<br>- Apex-forming LV.<br>- Left component of the common	1. Yes<br>2. Yes<br>- However, biventricular outcome not possible	No

© 2021 Salehi D et al. *JAMA Network Open.*

	- Left SVC.<br>- Interrupted IVC.<br><br>(Poor acoustic windows, progressive increase in ventricular unbalance)		→ Biventricular circulation possible.	AV valve with z score -2.7.<br>- Structural left AV valve abnormality.<br>- Aortic valve hypoplasia.<br>- CoA<br>- Interrupted IVC.<br>- Left SVC.<br>(Univentricular outcome)	mainly due to structural left AV valve abnormality but also due to aortic valve hypoplasia.	
15	Suspected aortic valve stenosis (narrow annulus, thickened valve).	1. Aortic valve annulus?<br>2. MV annulus size (uni- vs biventricular outcome)?	1. Normal-sized aortic valve annulus.<br>2. MV annulus not visualized.	- Normal-sized aortic valve.	1. Yes<br>2. –	No

© 2021 Salehi D et al. *JAMA Network Open.*

	- Borderline LV.<br>- No aortic arch hypoplasia or CoA.		→ Unable to predict uni- vs biventricular outcome.	- No aortic arch hypoplasia or CoA.<br>- Slightly underdeveloped MV annulus (Biventricular outcome).		
16	- PA-IVS<br>- Challenging fetal position in late pregnancy.	1. Tricuspid valve annulus size?<br>2. RV-anatomy?<br>(uni-vs biventricular outcome?)	1. Tricuspid valve annulus (z score -2.7).<br>2. Bipartite RV.<br><br>→ Likely biventricular outcome.	- PA-IVS<br>- TV annulus (z score -2.1).<br>- Hypoplastic, bipartite RV.<br>- Normal pulmonary valve annulus.	1. Yes<br>2. Yes	Improved counseling regarding likely biventricular outcome.

© 2021 Salehi D et al. *JAMA Network Open.*

				- No RV dependent coronary circulation demonstrated during postnatal cardiac catheterization.<br><br>(Biventricular outcome)		
17	- Borderline LV with suspected marked underdevelopment of the MV annulus	1. MV annulus diameter? (uni- vs biventricular outcome?)	1. Not visualized.<br><br>→ Unable to predict uni- vs biventricular outcome.	- Slightly underdeveloped left-sided cardiac structures.	1. –	No change in patient management (Uncertainty regarding uni- vs biventricular outcome).

© 2021 Salehi D et al. *JAMA Network Open.*

	(suboptimal projection).<br>- Aortic arch hypoplasia and narrow isthmus (high suspicion of CoA).			- Bicuspid aortic valve.<br>- Arch hypoplasia.<br>- CoA<br><br>(Biventricular outcome)		
18	- Slight ventricular disproportion. No arch hypoplasia but contraductal shelf (suspected CoA).	1. Aortic arch anatomy (hypoplasia/CoA)?	1. No aortic arch hypoplasia or CoA.	- No aortic arch hypoplasia or CoA.	1. Yes	Counseling<br><br>Delivery in hospital closest to patient's home possible option.
19	- Slight ventricular disproportion.	1. Aortic arch anatomy (hypoplasia/isthmic size): CoA?	1. No aortic arch hypoplasia, nomal-sized isthmus.	- Normal size of left-sided cardiac structures.	1. Yes	Counseling and planning of delivery.

© 2021 Salehi D et al. *JAMA Network Open.*

	- Aortic valve, ascending aorta and aortic arch slightly underdeveloped.<br>- Suspected narrowing in isthmic region.		→ Low risk of postnatal CoA.	- No arch hypoplasia.<br>- Normal-sized aortic isthmus.		Delivery in hospital closest to patient's home possible option.
20	- Unbalanced AVSD.<br><br>(Poor acoustic windows due to late pregnancy and maternal obesity)	1. Degree of ventricular unbalance (uni- vs biventricular outcome?)<br>2. CoA?<br>3. VSD?	1. Mildly unbalanced AVSD with RV dominance.<br>2. Marked aortic arch hypoplasia.<br>3. No large VSD component.	- Mildly unbalanced AVSD with RV dominance.<br>- Left component of the common AV valve with z score -2.2.	1. Yes<br>2. Yes<br>3. Yes	Counseling regarding CoA, confirmation of need of delivery at surgical center with prostaglandins available.

© 2021 Salehi D et al. *JAMA Network Open.*

			- LV apex-forming with 2 papillary muscles.<br><br>→ Biventricular outcome likely. High risk of CoA/need for aortic surgery.	- Almost apex-forming LV with preserved function.<br>- Small VSD.<br>- Aortic arch hypoplasia.<br>- CoA<br><br>(Biventricular outcome)		
21	- DORV-TGA<br>- Suspected aortic arch hypoplasia and narrow isthmus.	1. Aortic arch hypoplasia?<br>2. CoA?	1. Mild to moderate aortic arch hypoplasia.<br>2. Hypoplastic isthmus.	- DORV-TGA<br>- Aortic arch hypoplasia.<br>- CoA	1. Yes<br>2. Yes	Improved counseling regarding complexity and need for complicated biventricular repair.

	(Unable to visualize entire aortic arch)		→ High risk of postnatal CoA requiring arch reconstruction.			
22	- Mild LV hypoplasia.<br>- Mild aortic arch hypoplasia.<br>- Suspected narrowing of aortic isthmus.	1. Size of the aortic isthmus?	1. Narrow isthmus with potential postnatal development of CoA (z score -3.5).<br><br>→ May need postnatal CoA surgery.	- Slight narrowing in aortic isthmus.<br>- No CoA.<br>- No aortic arch hypoplasia.	1. Yes (narrowing but no CoA).	Echocardiography findings confirmed. No change in patient management.
23	- Mild LV hypoplasia.<br>- No aortic arch hypoplasia.<br>- Suspected narrow isthmus.	1. Aortic arch anatomy? (focus isthmic size)	1. No aortic arch hypoplasia. Slight narrowing of the aortic isthmus with low suspicion of postnatal CoA.	- Slight LV hypoplasia including MV annulus size.	1. Yes	Counseling: low suspicion of postnatal CoA.<br><br>No change in patient management.

© 2021 Salehi D et al. *JAMA Network Open.*

			→ Likely no need of postnatal intervention.	- No aortic arch hypoplasia or CoA.		
24	- Borderline LV with narrow MV annulus.<br>- Suspected slight arch hypoplasia with underdeveloped isthmus.<br><br>- Difficult to assess aortic arch (no sagittal arch view obtained).	1. Aortic arch anatomy? (hypoplasia, isthmic size)<br>2. Size of left-sided structures (MV annulus size + LV size/function)? (uni- vs biventricular outcome?).	1. No aortic arch hypoplasia, isthmus slightly hypoplastic (z score -2.1).<br>2. Mild LV hypoplasia (MV annulus size z score -3.4, narrow LV with good function).<br>→ Low risk of postnatal CoA. Biventricular outcome may be possible.	- No aortic arch hypoplasia.<br>- No CoA, but slight narrowing in isthmic region.<br>- Mild LV hypoplasia.<br><br>(Biventricular outcome)	1. Yes<br>2. Yes	Counseling: low suspicion of CoA, biventricular outcome.<br><br>Clinical assessment before discharge possible. No prostaglandins.

© 2021 Salehi D et al. *JAMA Network Open.*

25	- Borderline LV with suspected postnatal univentricular outcome.<br>- Uncertain if RAS.<br>- Pulmonary venous return to LA not visualized.<br>- Uncertain if aortic arch hypoplasia and/or CoA.	1. LV size and function.<br>2. MV annulus diameter (uni-vs biventricular outcome?).<br>3. Aortic arch anatomy (arch hypoplasia/isthmic size).<br>4. Pulmonary venous return to LA?<br>5. Interatrial communication:<br>a) Diameter (RAS?) and if small<br>b) Pulmonary lymphangiectasia?	1. Narrow LV with reduced lateral wall contractility.<br>2. MV annulus z score -5.4.<br>3. Aortic arch hypoplasia, isthmic z score -3, (high suspicion of CoA).<br>4. At least 2 pulmonary veins drain into LA.<br>5. No indirect signs of RAS. No pulmonary lymphangiectasia.	- Borderline LV with moderately underdeveloped left-sided cardiac structures.<br>- Aortic arch hypoplasia and CoA with need of arch repair.<br>- Bicuspid aortic valve.<br>- Normal pulmonary venous return to LA.	1. Yes<br>2. Yes<br>3. Yes<br>4. Yes<br>5. Yes	Parental counseling: uncertain uni- vs biventricular outcome; high likelihood for postnatal CoA repair; no RAS or lymphangiectasia or TAPVR (improved outcome).<br>No high-risk delivery. Vaginal delivery without cath lab on standby.

© 2021 Salehi D et al. *JAMA Network Open.*

	(Late referral from other center, very poor acoustic windows)		→ Uncertainty regarding uni- vs biventricular outcome. High risk of postnatal CoA.	(Biventricular outcome)		
26	- Borderline LV with suspected moderately underdeveloped mitral and aortic valve annulus (suboptimal projection).<br>- Suspected aortic arch hypoplasia	1. Aortic arch anatomy? (hypoplasia/isthmic size)<br>2. MV annulus size?<br>3. LV size and function? (uni- vs biventricular outcome?)	1. No arch hypoplasia, hypoplastic isthmus (z score -3) (suspicion of postnatal CoA).<br>2. MV annulus mildly underdeveloped (not optimally visualized).<br>3. Near apex-forming LV with good function.	- Mildly underdeveloped left-sided cardiac structures.<br>- Mild aortic arch hypoplasia.<br>- Mild CoA (follow-up: no operation yet).	1. Yes (mild CoA: but no need of postnatal operation).<br>2. Yes but not optimally visualized.<br>3. Yes	Parental counseling: high likelihood of biventricular outcome; postnatal CoA likely.<br><br>No change in postnatal clinical assessment.

© 2021 Salehi D et al. *JAMA Network Open.*

	and isthmal narrowing.		→ Biventricular outcome likely.	(Biventricular outcome)		
27	- Borderline LV with moderately hypoplastic mitral and aortic valve annulus: uncertain postnatal uni- vs biventricular outcome.<br>- Aortic arch hypoplasia.<br>- Suspected CoA.	1. MV annulus size (uni- vs biventricular outcome)?<br>2. CoA?	1. MV annulus mildly hypoplastic (z score -2.2).<br><br>→ Biventricular outcome likely.<br><br>2. Isthmus not optimally visualized, sagittal arch not visualized.<br><br>→ Unable to assess risk of CoA.	- Mild LV hypoplasia.<br>- Aortic arch hypoplasia with CoA.<br><br>(Biventricular outcome)	1. Yes<br>2. –	Counseling concerning risk of CoA but with biventricular outcome.

© 2021 Salehi D et al. *JAMA Network Open.*

28	- HLHS<br>- Unclear if RAS.<br>- Unable to visualize aortic arch.<br><br>(Poor acoustic windows)	1. Restrictive atrial communication?<br>2. Pulmonary lymphangiectasia?<br>3. Aortic arch anatomy?	1. Atrial communication with diameter 3 mm and left-to-right shunt.<br>2. No Pulmonary lymphangiectasia.<br>3. Aortic arch hypoplasia.<br><br>→ Low risk of restrictive atrial septum.	- HLHS without RAS.<br>- Aortic arch hypoplasia.	1. Yes<br>2. Yes<br>3. Yes	Parental counseling.<br><br>Standard-risk HLHS: vaginal delivery without cath lab on standby.
29	In another center suspected:<br>- Unbalanced AVSD with left dominance.	1. Degree of ventricular unbalance? (uni- vs biventricular outcome?)<br>2. Underdeveloped PV annulus?	1. Complete AVSD with slight unbalance (favoring the left).<br>2. PV annulus almost normal-sized.<br>3. Normal-sized MPA.	- Slightly unbalanced AVSD (left dominance).<br>- No pulmonary atresia.	1. Yes<br>2. Yes<br>3. Yes<br>4. –<br>5. –	Counseling: biventricular outcome likely.<br><br>No change in patient management because

© 2021 Salehi D et al. *JAMA Network Open.*

	- Pulmonary atresia with underdeveloped pulmonary branches.<br><br>(poor acoustic window due to late gestation and high BMI)	3. Underdeveloped pulmonary trunk?<br>4. And pulmonary branch arteries?<br>5. Pulmonary atresia?<br>(need of prostaglandins?)	4. Pulmonary branch arteries not visualized.<br>5. Flow from RV to MPA could not be confirmed.<br><br>→ Biventricular outcome likely.	- Well developed MPA and pulmonary branch arteries.<br><br>(Biventricular outcome)		pulmonary atresia could not be ruled out prior to delivery (otherwise no prostaglandins stand-by).
30	- Heart not visualized due to high maternal BMI and anterior placenta.	1. Basic anatomical and functional assessment to rule out major CHD.	1. Normal-sized ventricles with normal systolic function. No inflow anomalies.<br>No anomalies of outflow tracts.	- Normal cardiovascular anatomy and function.	1. Yes	Allows for delivery at hospital closest to patient's home with non-urgent postnatal assessment.

© 2021 Salehi D et al. *JAMA Network Open.*

	- Multiple risk factors for CHD.		No anomalies of great vessels.<br>One (possibly two) visible pulmonary vein to LA.			
31	- Univentricular heart with good single ventricular function (suspected HLHS).<br>- Impossible to visualize outflow tracts, atrial septum or connection of	1. Type of univentricular heart?<br>2. Pulmonary veins to LA?<br>3. RAS/IAS?<br>4. Pulmonary lymphangiectasia?<br>5. Anatomy and connection of outflow tracts?<br>6. Anatomy of aortic arch?	1. HLHS with narrow LV and VSD.<br>2. At least two pulmonary veins connected to LA.<br>3. Adequate interatrial communication.<br>4. No pulmonary lymphangiectasia.<br>5. Moderately hypoplastic aorta	- HLHS with narrow LV (aortic stenosis, mitral atresia and VSD).<br>- Normally connected pulmonary veins to LA.<br>- No RAS/IAS.<br>- Moderately hypoplastic aortic	1. Yes<br>2. Yes<br>3. Yes<br>4. Yes<br>5. Yes<br>6. Yes	Standard risk HLHS: vaginal delivery without cath lab on standby.<br><br>Improved counseling.

© 2021 Salehi D et al. *JAMA Network Open.*

	pulmonary veins to LA. (Poor acoustic windows due to high maternal BMI)		connected to the LV and dilated pulmonary artery connected to the RV. 6. Moderately hypoplastic aortic arch with posterior shelf. 7. Extracardiac malformations.	valve and aortic arch with posterior shelf. - Extracardiac malformations (VACTERL association).		

Supplementary Table 1 shows prenatal echocardiography diagnosis, diagnostic questions included in the fetal CMR referral, information obtained from fetal CMR and how this compared to postnatal diagnosis. For clarity, the second to last column lists to what extent fetal CMR was correct compared with postnatal diagnoses, and the last column lists impact on patient care. *AV=atrioventricular; AVSD=atrioventricular septal defect; BMI=body mass index; CHD=congenital heart defect; CMR=cardiovascular magnetic resonance; CoA=Coarctation of the aorta; DILV=double-inlet left ventricle; DORV=double-outlet right ventricle; HLHS=hypoplastic left heart syndrome; IVC=inferior vena cava; LA=left atrium; LV=left ventricle; MPA=main pulmonary artery; MV=mitral valve; PA-IVS=pulmonary atresia with intact ventricular septum;*

© 2021 Salehi D et al. *JAMA Network Open.*

PS=pulmonary stenosis; PV=pulmonary valve; RAS=restrictive atrial septum; RV=right ventricle; SVC=superior vena cava; TAPVR=total anomalous pulmonary venous return; TGA=transposition of the great arteries; VACTERL=vertebral defects, anal atresia, cardiac defects, tracheo-esophageal fistula, renal anomalies, and limb abnormalities; VSD=ventricular septal defect

© 2021 Salehi D et al. *JAMA Network Open.*
